# Tartrazine Removal from Aqueous Solution by HDTMA-Br-Modified Colombian Bentonite

**DOI:** 10.1155/2019/2042563

**Published:** 2019-09-08

**Authors:** Ronald A. Otavo-Loaiza, Nancy R. Sanabria-González, Gloria I. Giraldo-Gómez

**Affiliations:** ^1^Departamento de Ingeniería Química, Facultad de Ingeniería y Arquitectura, Universidad Nacional de Colombia Sede Manizales, Campus La Nubia, km 7 Vía al Aeropuerto, AA 127 Manizales, Colombia; ^2^Departamento de Física y Química, Facultad de Ciencias Exactas y Naturales, Universidad Nacional de Colombia Sede Manizales, Campus La Nubia, km 7 Vía al Aeropuerto, AA 127 Manizales, Colombia

## Abstract

The effect of pH, ionic strength (NaCl added), agitation speed, adsorbent mass, and contact time on the removal of tartrazine from an aqueous solution, using an organobentonite, has been studied. A complete factorial design 3^2^ with two replicates was used to evaluate the influence of the dye concentration (30, 40, and 50 mg/L) and amount of adsorbent (25, 35, and 45 mg) on decolorization of the solution. Experimental data were evaluated with Design Expert® software using a response surface methodology (RSM) in order to obtain the interaction between the processed variables and the response. pH values between 2 and 9, stirring speed above 200 rpm, and contact time of 60 min did not have a significant effect on decolorization. The optimum conditions for maximum removal of tartrazine from an aqueous solution of 30 mg/L were follows: pH = 6.0, NaCl concentration = 0.1 M, stirring speed = 230 rpm, temperature = 20°C, contact time = 60 min, and the organobentonite amount = 38.04 mg. The equilibrium isotherm at 20°C was analyzed by means of the Langmuir and Freundlich models, and the maximum adsorption capacity obtained was 40.79 ± 0.71 mg/g. This adsorption process was applied in a sample of industrial wastewater containing tartrazine and sunset yellow, having obtained a decolorization rate higher than 98% for both dyes. These results suggest that organobentonite is an effective adsorbent for the removal of anionic dyes from an aqueous solution.

## 1. Introduction

Organic pollutants commonly found in the aquatic environment are dyes, biocides compounds, phenols, surfactants, pesticides, and pharmaceuticals, among others [1, 2]. As coloring agents, some dyes are resistant to degradation and their presence in water might be harmful to human beings and hazardous to aquatic organisms [3].

A recent report on artificial food colors in grocery products marketed to children in North Carolina (USA) found that tartrazine was present in 20.5% of the products for consumption [4]. Tartrazine is a synthetic azodye used as a food colorant to achieve yellow or green shades in sweets, jellies, juices, jams, mustard, and sodas [5]. Additionally, it has been extensively used to dye human pharmaceuticals, such as vitamin capsules, antacids, and cosmetics [5, 6]. Pharmaceutical manufacturers and distributors from Canada indicate that approximately 450 products contain tartrazine [7].

Toxicokinetic studies showed that less than 2% of ingested tartrazine is directly absorbed and most tartrazine is broken down into metabolites such as sulfanilic acid and aminopyrazolone in the colon [8, 9]. A recent study showed clear absence of genotoxic activity for tartrazine in the bone marrow micronucleus assay and the Comet assay in the liver, stomach, and colon of mice [10]. However, as an azodye, tartrazine was subject to mutagenicity concern associated with the possible generation of free amines in vivo by azoreduction [11]. Other studies have reported that tartrazine may cause allergic reactions to some people, specifically those with asthma or aspirin intolerance [12]. It has been suggested that children with hyperactivity can develop increased irritability, restlessness, and sleep disturbances after taking tartrazine [13].

The high consumption of tartrazine is also guilty for high amounts of this dye to be lost during the processing of food and medicines, generating an environmental problem. Dyes are visible to the human eye, even in a low concentration (<1 mg/L) [1, 14], and can reduce photosynthetic activity in aquatic environments by preventing the penetration of light and oxygen [15, 16]. Therefore, food industries must treat their dye-containing effluents before discharging them into natural water sources.

Among various water treatment methods such as ion exchange, coagulation/flocculation, membrane filtration, reverse osmosis, chemical precipitation, advanced oxidation, and biological processes [3, 15, 17–20], adsorption is one of the most widely used methods for the removal of dyes due to simplicity of design, low cost, easy operation, high efficiency, and reusability of material [20–25].

Various naturally occurring materials have been explored as adsorbents for the removal of dyes from wastewater [26–28], activated carbon being one of the most effective; however, it is not frequently used due to its high cost [29]. As an alternative to activated carbon, organic materials obtained from living or dead creatures (biomass) and agriculture waste have been used as low-cost adsorbents for the removal of pollution [20, 25, 30].

Natural clays are considered to be excellent low-cost adsorbents for the removal of dyes from aqueous solutions due to their high surface area and porosity, layered structure, and high cation exchange capacity [31]. They include bentonite, montmorillonite, perlite, dolomite, illite, sepiolite, and kaolinite. The adsorption efficiency of clays is considerably enhanced after chemical modification through acid activation, thermal activation, intercalation and pillaring, surfactant treatment, and coating with metal oxide and clay composites made with different biopolymers [3, 24, 32].

One of the most commonly used clays as an adsorbent is montmorillonite, a clay mineral member of the smectite group. The smectite layer structure is composed of two tetrahedral sheets packed in an octahedral sheet to form a TOT or 2 : 1 layer [33, 34]. Isomorphic substitutions by lower-valence cations occurring in tetrahedral and/or octahedral sheets induce a net negative charge of the layer. This deficit is compensated by the presence of exchangeable cations in the interlayer space (commonly Na^+^, K^+^, and Ca^2+^). Although the adsorption capacity of montmorillonite for cations is very high, it is low for anions. This situation can be modified through an exchange of the inorganic cations with organic cations (surfactants), resulting in an organically modified clay mineral (so-called organoclay) [34–36]. The most common cationic surfactants used for clay modification are quaternary ammonium salts, such as HDTMA (hexadecyltrimethylammonium bromide), ODTMA (octadecyltrimethylammonium bromide), and TMAB (tetradecyltrimethylammonium bromide) [3]. This organically modified clay mineral has a high adsorption capacity for anionic dyes [37], acid dye (for example, methyl orange and acid red) [38, 39], and reactive dyes (such as remazol brilliant blue R) [40].

Raw bentonite possesses good adsorption properties of cationic dyes, but low adsorption affinity of anionic dyes (as tartrazine). Therefore, in this study, bentonite was modified with a cationic surfactant to incorporate positive charge sites for the adsorption of anionic species. Studies on the removal of tartrazine from a solution using a modified bentonite are limited [41]. In Colombia, it has been estimated that smectite clay deposits for exploitation are 1.1 × 10^9^ metric tons [42], which could be used as an adsorbent material. This study evaluates the effect of variables pH, ionic strength, agitation speed, adsorbent mass, and contact time on the adsorption of a tartrazine dye in an aqueous medium, using a Colombian bentonite modified with hexadecyltrimethylammonium bromide (HDTMA-Br) as the material adsorbent. Additionally, the effectiveness of the adsorbent to remove anionic dyes with a sample of wastewater from a local food industry was evaluated.

## 2. Experimental

### 2.1. Reagents and Materials

The azodye tartrazine (C_16_H_9_N_4_Na_3_O_9_S_2_, 534.3 g/mol, CAS registry number: 1934-21-0) used in this study was of consumer quality (purity 62%, 38% NaCl and NaSO_4_ combined) purchased from Retema S.A.S.-Colombia, without further purification. The stock solution (100 mg/L) was prepared by accurately dissolving a weighed quantity of the dye in double-distilled water. An experimental dye solution in different concentrations was arranged by diluting the stock solution into a suitable volume of double-distilled water.

The surfactant hexadecyltrimenthylammonium bromide (denoted as HDTMA-Br, molecular formula: CH_3_(CH_2_)_15_N(Br)(CH_3_)_3_, 364.46 g/mol, CAS registry number: 57-09-0, purity >98.0%) was purchased from Panreac®.

The clay used was a Colombian bentonite sample from Armero-Guayabal municipality in the north of the department of Tolima. The mineralogical and chemical composition of this bentonite has been previously reported [42]. The quantitative study for the mineral composition of the bentonite shows a content of montmorillonite (48%), quartz (21%), and plagioclase (11%). The separation by particle size of the clay fraction (<2 *μ*m) was made by gravitational sedimentation and then, the purified bentonite was converted to sodium bentonite (denoted as Na-Bent) by two exchanges with the NaCl solution. The Na-Bent obtained was repeatedly washed with distilled water until the leachate showed a negative test for chloride ions, dried at 60°C and, finally, ground and sieved in a 100-mesh.

The cation exchange capacity (CEC) of Na-Bent was 41.16 meq/100 g, determined by using the ammonium acetate method [43]. The chemical composition of the sodium bentonite (Na-Bent) obtained by XRF was 55.35% SiO_2_, 16.28% Al_2_O_3_, 7.50% Fe_2_O_3_, 2.24% MgO, 1.11% CaO, and 3.43% Na_2_O.

### 2.2. Synthesis and Characterization of the Organobentonite

The total amount of the cationic surfactant used in the modification of the bentonite was 1.5 times the value of CEC, a value within the range recommended in the literature [44–46]. The modification included two steps. In the first step, the sodium bentonite was suspended in water (50 g Na-Bent in 1 L of distilled water) and left to stir for 12 h to achieve swelling. In the second step, a quaternary ammonium cation solution (12 g of HDTMA-Br in 500 mL of distilled water) was slowly added into the suspension containing the Na-Bent and vigorously stirred for 24 h. The treated bentonite was separated from the suspension by centrifugation at 5000 rpm and repeatedly washed until a negative bromide test with 0.1 M of AgNO_3_ was obtained. The washed organobentonite was then dried in an oven at 80°C for 12 h and subsequently at 100°C for 2 h. Finally, the organobentonite (denoted as HDTMA-Bent) was ground to obtain a particle size of 100 mesh.

Na-Bent and HDTMA-Bent were both characterized by X-ray diffraction (XRD), content of total organic carbon (TOC), Fourier transform infrared spectroscopy (FT-IR), and nitrogen adsorption at 77 K. The diffraction patterns were taken to a LabX Shimadzu XRD-6000 diffractometer with Cu K*α* radiation (steps of 0.02 2 *θ* and 2 s/step). Total organic carbon was estimated by Multi N/C 3100 TOC analyzer (Analytik Jena, Germany) in a horizontal high-temperature oven HT1300 for solid sample analysis, equipped with a nondispersive infrared detector. The calibration was made with analytical-grade CaCO_3_. The calculated error for this technique was ±0.20%. Fourier transform infrared spectrometry (FT-IR) was recorded from samples pressed into pellets with KBr powder by using a Nicolet iS5 (Thermo Scientific). Nitrogen adsorption-desorption isotherms were determined in a Micromeritics ASAP 2020 instrument at 77 K after outgassing the samples for 3 h at 90°C, followed by 2 h at 150°C in a vacuum.

### 2.3. Batch Adsorption Experiments

In the present study, batch adsorption experiments were carried out at ambient conditions (20°C and atmospheric pressure). For this, 50 mL solution of tartrazine at 20 mg/L were put into 100 mL Erlenmeyer flasks and an amount of organobentonite added to the solutions. The mixture was magnetically shaken at a constant speed using a 5-position digital magnetic hotplate stirrer (RT 5, IKA, Germany). The methodological design to evaluate the adsorption of tartrazine independently analyzed the effect of each factor (pH, ionic strength, agitation speed, adsorbent mass, and contact time), keeping the other parameters constant, as shown in Table 1. The pH of the solution was adjusted with 0.1 N HCl and NaOH solutions, and monitored with an SI Analytics Lab 845 pH meter. The effect of the ionic strength on the adsorption process was evaluated at different concentrations of NaCl in the solution of tartrazine.

The dye concentration was determined from aliquots (1 mL of sample filtered in 0.45 *μ*m millipore paper), while the percentage of decolorization was established as a response variable (equation (1)), quantifying the dye concentration from a previous calibration curve, obtained by UV-Vis spectrophotometry (Mapada V-1200) at a wavelength (*λ*) of 428 nm:

(1) $$\begin{array}{c} \text{decolorization }\left(\%\right)=\;\frac{C_{o}-C_{t}}{C_{o}}\times 100, \end{array}$$

where *C*_*o*_ and *C*_*t*_ (mg/L) are the liquid-phase concentrations of the dye at initial and any time *t*, respectively. All tests were performed in triplicate.

### 2.4. Experimental Design and Adsorption Isotherm

Based on the initial test of the methodological design (Table 1), the variables that mainly affect the adsorption process were defined, and an experimental design was established to determine the optimal conditions of adsorption.

The amount of tartrazine adsorbed at 20°C was evaluated at different initial concentrations of tartrazine ranging from 30 to 100 mg/L at the optimal conditions obtained from the experimental design. The adsorption capacity (*q*_*e*_, mg/g) of dye was calculated using the following equation:

(2) $$\begin{array}{c} q_{e}=\;\frac{C_{o}-C_{e}}{W}\times V, \end{array}$$

where *C*_*o*_ and *C*_*e*_ are the initial and equilibrium concentrations of the dye (mg/L), respectively, *V* is the volume (L), and *W* is the mass (g) of the adsorbent.

Adsorption isotherm data were fitted by the Freundlich and Langmuir models, which are the most frequently used. The Langmuir model assumes that there is no interaction between the adsorbate molecules and that the adsorption takes place in a monolayer [47, 48]. The Langmuir isotherm is represented by the following equation:

(3) $$\begin{array}{c} \;q_{e}=\frac{Q_{\max}K_{\text{L}}C_{e}}{1+K_{\text{L}}C_{e}}, \end{array}$$

where *q*_*e*_ is the adsorbate equilibrium amount in the solid phase (mg/g), *C*_*e*_ is the adsorbate equilibrium concentrations in solution (mg/L), *Q*_max_ is the maximum adsorption capacity according to Langmuir monolayer adsorption (mg/g), and *K*_L_ is constant according to the Langmuir model (L/mg).

The Freundlich isotherm model is an empirical relationship describing the adsorption of solutes from a liquid to a solid surface, and it assumes that different sites with several adsorption energies are involved [48, 49]. The form of the Freundlich equation is as follows:

(4) $$\begin{array}{c} q_{e}=K_{\text{F}}C_{e}^{1/n}, \end{array}$$

where *K*_F_ (L/g) and *n* are the Freundlich constants related to the adsorption capacity and adsorption intensity of the adsorbent, respectively.

### 2.5. Batch Adsorption Experiment with a Sample of Wastewater

The applicability of the adsorbent was evaluated in a sample of industrial wastewater taken from the washing process of a local industry in the food sector. The specifications given by the supplier of the sample correspond to an effluent rich in sugars, dyes (tartrazine and sunset yellow), and other compounds, with pH 3.5. Once the sample was obtained, a UV-Vis spectrum was performed to identify the wavelengths of maximum absorption, associated with the chromophore groups present in the sample.

## 3. Results and Discussion

### 3.1. Characterization of the Clay and Organoclay

**Figure 1:**
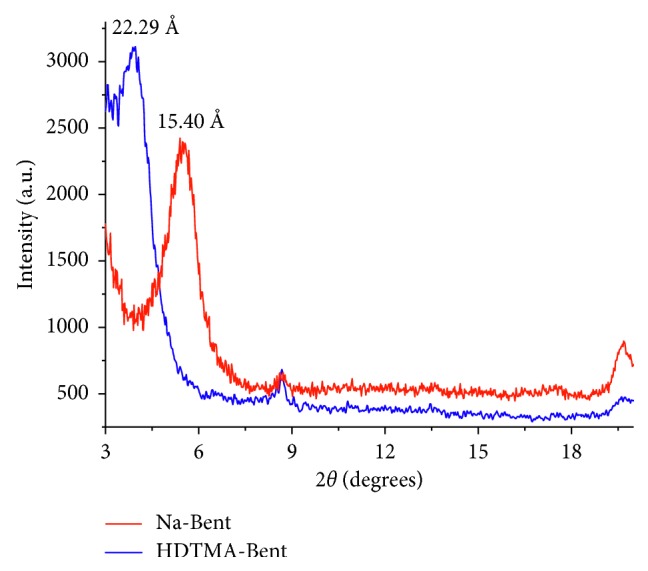
XRD patterns of sodium bentonite and organobentonite.

Figure 1 shows the diffraction patterns of sodium bentonite and organobentonite. Na-Bent has a basal spacing *d*_001_ at 15.4 Å, characteristic of a montmorillonite and, in HDTMA-Bent, it is increased to 22.3 Å. The intercalation of the HDTMA^+^ cations tends to maximize their contact with the silicate surface and, hence, the basal spacing increases as more quaternary ammonium cations are accommodated in the interlayer spaces [50]. Basal spacing of 21.7 Å indicates that HDTMA^+^ cations may intercalate as a pseudotrimolecular layer, while the basal spacing >22.0 Å is related to a paraffin-like arrangement, including tightly packed molecules inclined at a high angle towards the interlayer surface, with quaternary ammonium cations lying parallel to one another [51, 52]. The basal spacing of 22.3 Å obtained for organobentonite can be associated with the incorporation of the HDTMA^+^ cation in a pseudotrilayer/paraffinic arrangement [53, 54]. This was due to the charge heterogeneity of clay mineral layers [55].

The measured total organic carbon content of Na-Bent and HDTMA-Bent provided a measure of the quantity of quaternary organic cation intercalated into organoclay. The TOC for Na-Bent was 93.82 ± 0.49 mg of C/kg, a very low value that suggests a minimum amount of organic matter in the sample. The HDTMA-Bent showed a TOC of 113.85 ± 1.85 g of C/kg because the exchanged organic surfactant contained carbon in its structure. The incorporation of the HDTMA^+^ cation in the clay was 77.68 ± 1.30%.

**Figure 2:**
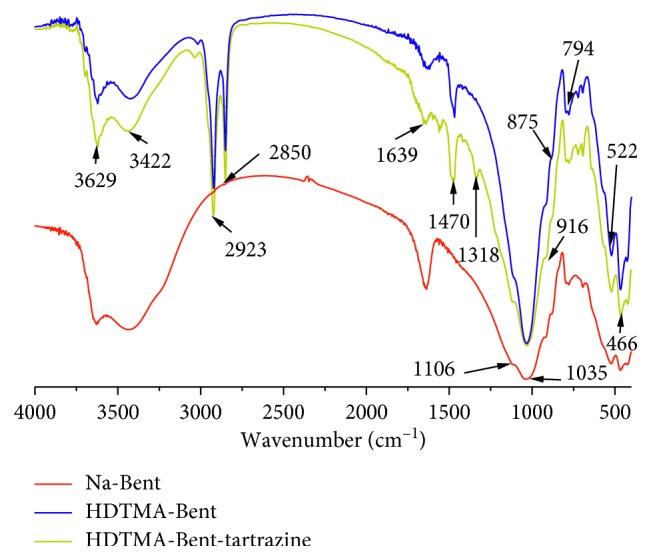
FT-IR spectra of sodium bentonite and the organobentonite before and after adsorption of tartrazine.

Figure 2 shows the FT-IR spectra for Na-Bent and HDTMA-Bent. The two spectra show the presence of -OH as stretching bands at 3629 cm^−1^ as well as bending bands at 916 cm^−1^. The large band at 1035 cm^−1^ corresponds to the Si-O stretching vibration. Above signals can be considered characteristic of dioctahedral smectite [42, 56]. Broad bands centered near 3422 and 1639 cm^−1^ are due to the –OH stretching mode of the interlayer water. The overlaid absorption peak in the region of 1639 cm^−1^ is assigned to the -OH bending mode of adsorbed water [42, 57]. The band in the region of 875 cm^−1^ is due to the Si-O-Al stretching mode for montmorillonite [57, 58]. The bands at 794 and 1037 cm^−1^ correspond to Si-O stretching vibration of quartz [59]. The bands at 522 cm^−1^ and 466 cm^−1^ are assigned to the Si-O-Al and Si-O-Si bending vibration, respectively [57, 58, 60].

In the organobentonite, a pair of strong bands at 2850 and 2923 cm^−1^ was observed. These are assigned to the symmetric and asymmetric stretching vibrations of the methylene groups (νCH_2_) and their bending vibration at 1470 cm^−1^ [61]. Previous bands are characteristic of bentonite modified with HDTMA [61, 62].

**Figure 3:**
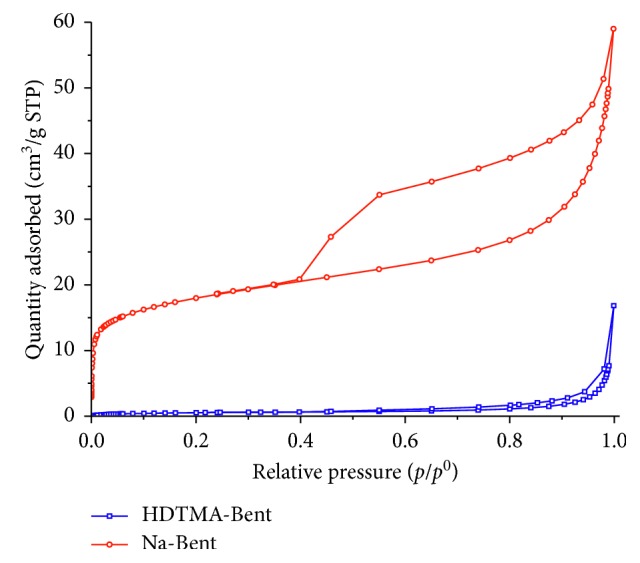
Nitrogen adsorption/desorption isotherms of sodium bentonite and organobentonite.

The adsorption-desorption isotherms of N_2_ at 77 K for Na-Bent and HDTMA-Bent are shown in Figure 3. Na-Bent presented an isotherm type IVa with an H3 hysteresis loop. Type IVa isotherm is characteristic of mesoporous adsorbents, while H3 hysteresis loops are associated with nonrigid aggregates of plate-like particles [63]. The HDTMA-Bent isotherm could be a combination of type III and IVa, with a small hysteresis loop H3, characteristic of nonporous or macroporous solids.

The specific surface area calculated with the BET model for the Na-Bent was 62.9 m^2^/g, of which 16.5 m^2^/g corresponds to the micropore area. The low specific surface area of the organobentonite (3.2 m^2^/g) indicates that only a part of the surface was accessible to the nitrogen gas [64]. Jacobo-Azuara et al. found that the specific surface area of an organobentonite (bentonite from a deposit of San Luis Potosi, Mexico, modified with HDTMA) was 2 m^2^/g [65], similar to the value obtained in this study. Due to the intercalation of the HDTMA^+^ cations in the interlaminar space of the bentonite in a paraffinic configuration, the pores are covered and there is a blockade that inhibits the passage of N_2_ molecules [44, 66].

### 3.2. Batch Adsorption Experiments

**Figure 4:**
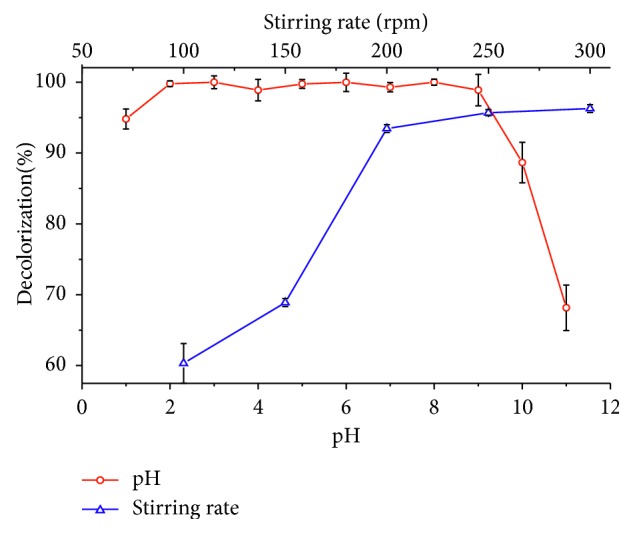
Effect of pH and the stirring rate on the adsorption of tartrazine on organobentonite.

Figure 4 presents the results of the effect of pH and agitation speed on the adsorption of tartrazine, using HDTMA-Bent. Decolorization of the solution is not significantly affected in the range of pH from 2 to 9, where the average removal was 99.07 ± 0.93%, this being a favorable aspect for the application of this material since the colored wastewater has pH values near 7 [67]. For a pH > 9, a negative effect on the removal of tartrazine was observed because, at high pH values, the electrostatic repulsion between the surface of the adsorbent, negatively charged, and the anionic dye reduces the adsorption capacity and removal of coloring [68, 69]. Test of adsorption with Na-Bent in the same range of pH showed no removal of color, having been 6.0 ± 0.8% at pH 2, the maximum value reached (results not shown in this work).

Less than 200 rpm shaking speeds (Figure 4) fail to thoroughly mix the adsorbent with the solution, while 250 and 300 rpm agitation speeds got decolorization higher to 97.33 ± 0.49% and ensured uniformity in the medium [70]. Gautam et al. worked with similar agitation speeds (>180 rpm) for the adsorption of tartrazine on a copper coordinated dithiooxamide metal-organic framework (Cu-DTO MOF) [71].

**Figure 5:**
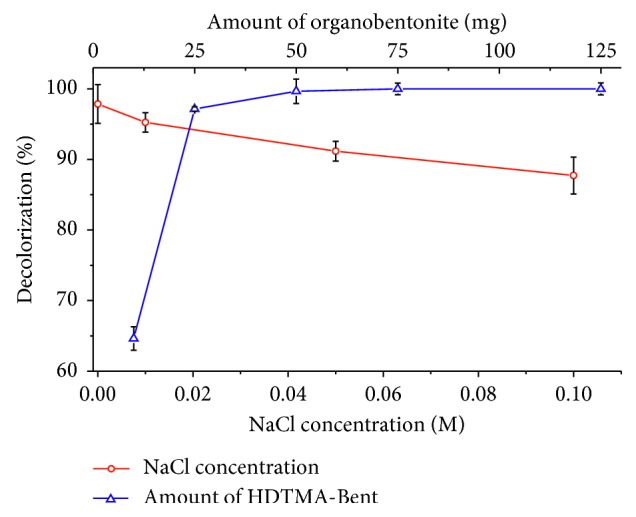
Effect of the addition of NaCl to the dye solution and amount of HDTMA-Bent on the adsorption of tartrazine.

The effect of the addition of NaCl and the amount of adsorbent in the removal of tartrazine on HDTMA-Bent are shown in Figure 5. The increase of the concentration of NaCl decreased the adsorption capacity of tartrazine from 97.85 ± 2.74% of decolorization, in the absence of NaCl, to 87.72 ± 2.62%, when the concentration of NaCl was 0.1 M. Monitoring of the ionic strength is important due to the fact that industrial wastewaters contain pollutants such as inorganic salts (NaCl, KCl, and CaCl_2_) [27] that affect the adsorption of acid dyes such as tartrazine. The reason is, mainly, a competition between the Cl^−^ ions and anions of tartrazine (C_16_H_9_N_4_O_9_S_2_^3−^) as well as the difference in size between these two ions, which makes the Cl^−^ ions to easily bind to the active sites of the adsorbent in comparison with the large-sized anions of tartrazine [71].

The effect of the amount of HDTMA-Bent in the decolorization of tartrazine is shown in Figure 5. For a mass of 10 mg of adsorbent, the decolorization reached was low (64.63 ± 1.66%) due to the rapid saturation and limited availability of active sites in the adsorbent [27]. For a mass of 25 mg HDTMA-Bent, the decolorization was 97.15 ± 0.30% and, with an amount higher than 50 mg, the removal was kept constant in a 99.67 ± 0.33%. The increase of the decolorization of tartrazine obtained with greater amounts of adsorbent is associated with the existence of a greater number of active sites for the removal of dye [27, 72].

**Figure 6:**
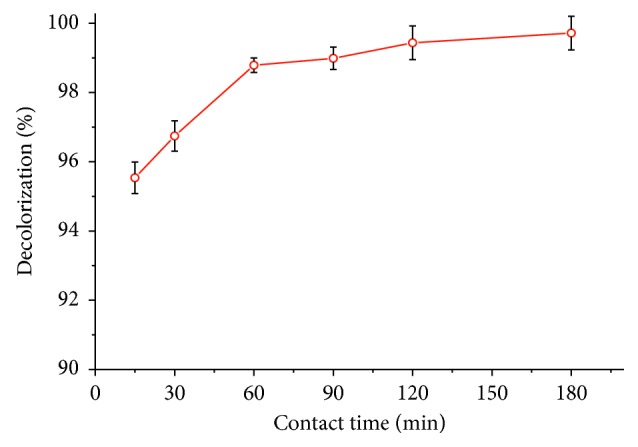
Effect of time contact on the adsorption of tartrazine on organobentonite.

Figure 6 presents the results of the contact time between the adsorbent and the solution of tartrazine. It was found that the adsorption process has two stages: the initial stage, which is rapid and occurs in the first 30 min, implying strong electrostatic attractions between tartrazine and the organobentonite; the second stage is slow, and the adsorption tends to become stable (in equilibrium) after 60 min since it shows no considerable change in the decolorization, implying the saturation of active sites in the HDTMA-Bent [27, 73].

The FT-IR spectrum of the adsorbent after the removal of tartrazine at 120 min shows an additional signal at 1318 cm^−1^ (Figure 2), which is associated with the C-N stretching of the structure of tartrazine [71].

From the analysis of the results of the parameters that affected the adsorption of tartrazine on the organobentonite, it was established that the main variables that affected the process were the amount of adsorbent and the addition of NaCl to the dye solution. pH values between 2 and 9, stirring speed above 200 rpm, and contact time of 60 min did not have a significant effect on decolorization.

### 3.3. Experimental Design

With the results of the initial tests of the methodological design (Table 1), a complete factorial design 3^2^ was established, having the amount of adsorbent (*A*) and the concentration of tartrazine (*B*) as variables. The levels for *A* and *B* were 25, 35, and 45 mg and 30, 40, and 50 mg/L, respectively. All the experiments were carried out in triplicate and were kept constant: pH = 6, contact time = 60 min, concentration of NaCl = 0.1 M, and agitation speed = 230 rpm. The response variable was decolorization percentage (%). Experimental data were evaluated with a Design Expert® software version 8.0 (StatEase, Inc., Minneapolis, MN, USA) using a response surface methodology (RSM) in order to obtain the interaction between the processed variables and the response. Data were adjusted to a second-order polynomial equation to determine the coefficients of the response model as well as their standard errors and significance [74]. For the two variable inputs under consideration, the response model is as shown in the following equation:

(5) $$\begin{array}{c} Y=\;\beta_{0}+\overset{k}{\underset{i=1}{\sum }}\beta_{i}X_{i}+\overset{k}{\underset{i=1}{\sum }}\beta_{ii}X_{i}^{2}\overset{k-1}{\underset{i=1}{\sum }}\overset{k}{\underset{\begin{array}{c} j=2\\ j>i \end{array}}{\sum }}\beta_{ij}X_{i}X_{j}, \end{array}$$

where *Y* is the predicted response (decolorization, %); *β*_0_, *β*_*i*_, *β*_*ii*_, and *β*_*ij*_ are the regression coefficients for the intercept and the linear, quadratic, and interaction coefficients, respectively; *X*_*i*_ and *X*_*j*_ are the independent variables, and *k* = 2, i.e., the number of independent variables. The quality of the model fits was evaluated by the coefficients of determination (*R*^2^ and adjusted *R*_adj_^2^) and analysis of variance (ANOVA).

Table 2 shows the codified and experimental values of the runs performed in the experimental design along with the response observed (average of the three repetitions).

The ANOVA results are shown in Table 3. The model and coefficients were considered significant for a *p* value < of 0.05.

Data in Table 3 indicate that the model and parameters are significant. The *p* values show that coefficients of the main effects are highly significant (*p* < 0.0001), compared to the interaction effect. The second-order response function representing the relationship between the decolorization (%) and the independent variables appears in the following equation:

(6) $$\begin{array}{c} \text{decolorization }\left(\%\right)=-137.17276\;+\;10.31623A\;+\;2.67123B\;-\;0.05194AB\;-\;0.09737A^{2}\;-\;0.03913B^{2}.\; \end{array}$$

All first-order coefficients of the model for the response decolorization showed positive effects, whereas the quadratic and interaction coefficients had a negative effect. Coefficients of determination (*R*^2^) and adjusted *R*_adj_^2^ of the model were 0.9710 and 0.9640, respectively, evidencing that this regression is statistically significant and that only 3.60% of the total variations are not explained by the model [70, 75].

**Figure 7:**
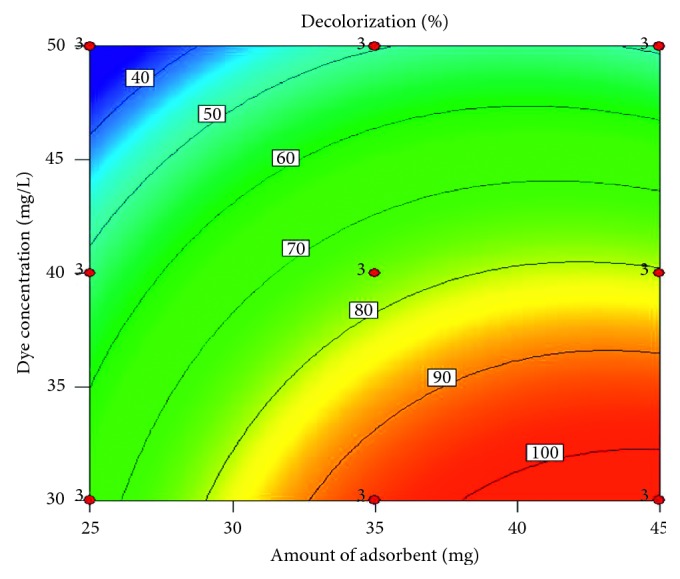
Contour plot for decolorization of tartrazine vs dye concentration and amount of adsorbent.

Figure 7 shows the contour plot that represents the interaction of the independent variables (dye concentration and amount of adsorbent). It illustrates that the decolorization has a tendency to decrease, when increasing the concentration of tartrazine and decreasing the amount of HDTMA-Bent. A region is also observed where the model establishes a complete decolorization when the dye concentration is lower than 33 mg/L and the amount of the adsorbent is greater than 37 mg.

To confirm the results obtained with the mathematical model presented in equation (6) of the experimental design, additional adsorption tests were carried out. The points were taken within the design range and classified as low, medium, and high with respect to the response (decolorization). The experiments were carried out in triplicate, at the same conditions in which the model was obtained (pH = 6, NaCl concentration = 0.1 M, contact time = 60 min, and agitation speed = 230 rpm).

From the results presented in Table 4, it is observed that, for the three points evaluated, the maximum difference between the experimental value and the one calculated with equation (6) was 4.8%. Therefore, the model obtained can be used to predict the response of the system with minimal variations.

**Figure 8:**
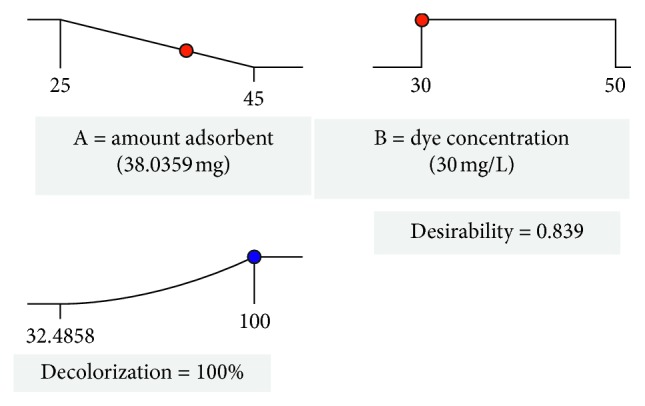
Desirability ramp for numerical optimization of decolorization.

A numerical optimization was performed for the factors and the response of the experimental design. The criteria selected in the software to perform the optimization were to minimize the amount of the adsorbent and maximize the decolorization. A higher degree of importance was assigned to the response because it is the main objective of the process.  Figure 8 shows the best scenario for the combined criteria in a function of global desirability, where the maximum decolorization (100%) is reached when the amount of adsorbent is 38.04 mg and the concentration of the dye is 30 mg/L, with a desirability of 0.839. The function of desirability varies between zero, which is outside the limit, and one, which is the goal, and indicates how close the lower and upper limits of the factors were established in relation with the actual optimum value [70].

**Figure 9:**
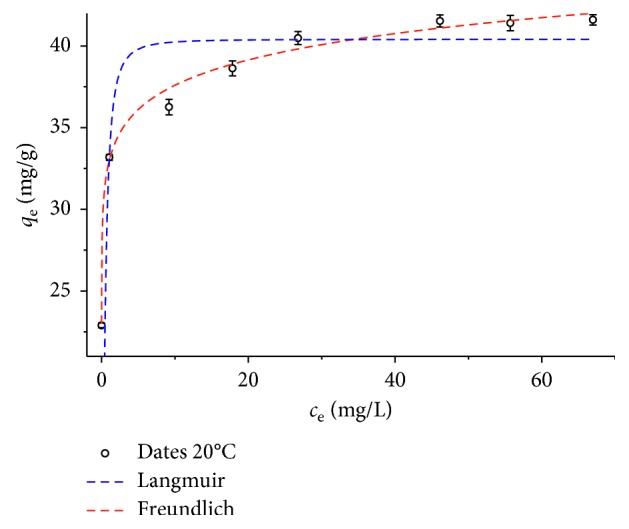
Adsorption isotherm of tartrazine onto HDTMA-Bent at 20°C.

The tartrazine adsorption isotherm at 20°C was evaluated at dye concentrations of 30, 40, 50, 60, 70, 80, 90, and 100 mg/L with an amount of organobentonite = 0.45 g and the same experimental conditions used for obtaining the model (*V* = 50 mL, pH = 6.0, NaCl concentration = 0.1 M, contact time = 60 min, and agitation speed = 230 rpm). The data obtained from the adsorption isotherm were fitted to the nonlinear form of Langmuir and Freundlich models using equations (3) and (4), respectively (Figure 9). Parameters of the fit were determined and are presented in Table 5.

The high value of the correlation coefficient obtained for the adsorption of tartrazine onto organobentonite indicates that the Freundlich model (*r*^2^ = 0.988) can be applied to this system better than the Langmuir model (*r*^2^ = 0.916), indicating a heterogeneous adsorption surface instead of a homogeneous monolayer adsorption as predicted by the Langmuir adsorption model. The *K*_F_ and *n* values calculated from the Freundlich isotherm were 33.02 ± 0.27 L/g and 17.64 ± 0.89, respectively. The high value of *n* confirms the heterogeneous adsorption system as predicted by the Freundlich adsorption model and the efficiency of HDTMA-Bent as a material adsorbent toward tartrazine removal. The maximum adsorption capacity of tartrazine obtained in this study was compared with that of other adsorbents in the literature, as shown in Table 6. The HDTMA-Bent showed a value of adsorption capacity similar to that of a bentonite modified with octadecyltrimethylammonium and superior to that of other low-cost adsorbents such as sawdust and chitin.

### 3.4. Batch Adsorption Experiment with a Sample of Wastewater

**Figure 10:**
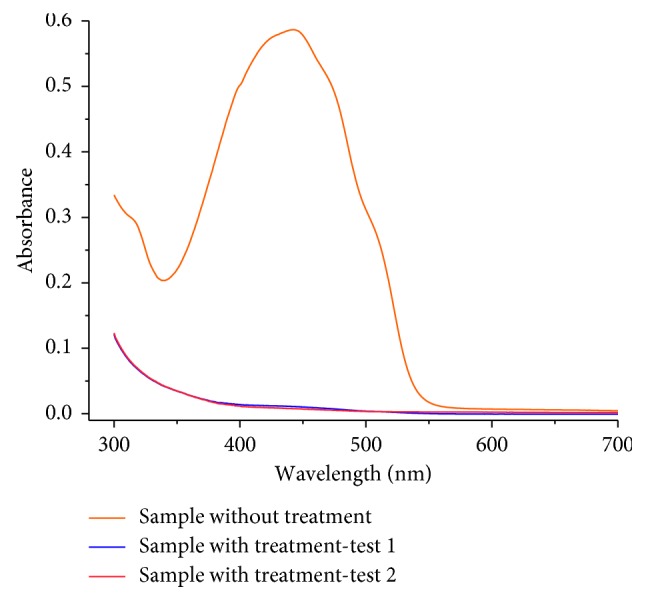
UV-Vis spectra of wastewater sample before and after adsorption.

Figure 10 showed the UV-Vis of the sample of the wastewater before and after the adsorption with Bent-HDTMA (duplicate). The test conditions were as follows: volume of solution = 50 mL, amount of adsorbent = 45 mg, pH = 6, contact time = 60 min, concentration of NaCl = 0.1 M, and agitation speed = 230 rpm. In the untreated wastewater sample, a pronounced and wide signal was observed, between 350 and 540 nm, corresponding to the maximum adsorption wavelengths of tartrazine (428 nm) and sunset yellow (482 nm). The average decolorization of tartrazine and sunset yellow was 98.19 and 98.76%, respectively. Considering that sunset yellow is an azodye (C_16_H_10_N_2_Na_2_O_7_S_2_), anionic, with characteristics similar to tartrazine, it is concluded that the Bent-HDTMA adsorbent was efficient in the removal of both dyes, although it was not selective.

## 4. Conclusions

In this study, a Colombian bentonite was modified with HDTMA-Br and used to remove tartrazine from an aqueous solution, obtaining high levels of decolorization, with the advantage of using a natural, abundant, and low-cost material.

It was established that the main variables that affect the adsorption of tartrazine were the amount of adsorption and the addition of NaCl to the dye solution. pH values between 2 and 9, stirring speed above 200 rpm, and contact time of 60 min did not have a significant effect on decolorization.

To achieve the total decolorization of a 30 mg/L tartrazine aqueous solution, 38.04 mg of HDTMA-Bent was required under the following conditions: pH = 6.0, NaCl concentration = 0.1 M, stirring speed = 230 rpm, temperature = 20°C, and contact time = 60 min.

## Figures and Tables

**Table 1:** The methodological design to evaluate the adsorption of tartrazine independently analyzed the effect of each factor.

Test	pH	Ionic strength (NaCl, M)	Agitation speed (rpm)	Adsorbent mass (mg)	Contact time (min)
1	1, 2, 3, 4, 5, 6, 7, 8, 9, 10, 11	0	300	25	60
2	6	0, 0.01, 0.05, 0.1	300	25	60
3	6	0	100, 150, 200, 250, 300	25	60
4	6	0	300	10, 25, 50, 75, 125	60
5	6	0	300	25	15, 30, 60, 90, 120, 180, 240

**Table 2:** Factorial design for the independent variables used in this study along with the observed response.

Test	Code values		Real values		Response
	*A*	*B*	*A* (mg)	*B* (mg/L)	Decolorization, (%)
1	−1	−1	25	30	63.72 ± 1.67
2	0	−1	35	30	99.99 ± 0.01
3	1	−1	45	30	99.99 ± 0.02
4	−1	0	25	40	50.36 ± 0.38
5	0	0	35	40	71.02 ± 0.68
6	1	0	45	40	87.59 ± 1.44
7	−1	1	25	50	32.82 ± 0.36
8	0	1	35	50	49.58 ± 0.84
9	1	1	45	50	46.50 ± 0.61

**Table 3:** Results of regression analysis (ANOVA).

Source	Sum of square	DF	Mean square	*F* value	*p* value
Model	13962.18	5	2792.44	140.40	<0.0001
*A*	3644.00	1	3644.00	183.22	<0.0001
*B*	9333.73	1	9333.73	469.30	<0.0001
AB	323.76	1	323.76	16.28	<0.05
*A* ^2^	568.82	1	568.82	28.60	<0.0001
*B* ^2^	91.87	1	91.87	4.62	<0.05

**Table 4:** Points for the validation of the experimental design.

Adsorbent (mg)	Dye concentration (mg/L)	Decolorization (%)		Error (%)
		Experimental	Calculated	
27	47.39	44.68 ± 0.34	42.64	4.79
40	43.74	69.15 ± 0.50	70.78	2.31
36	34.58	92.73 ± 0.14	88.93	4.27

**Table 5:** Equilibrium isotherm parameters for the adsorption of tartrazine onto organobentonite.

Langmuir	*Q*_max_ (mg/g)	40.79 ± 0.71
	*K*_L_ (L/mg)	4.06 ± 0.62
	*r* ^2^	0.916

Freundlich	*K*_F_ (L/g)	33.02 ± 0.27
	*n*	17.64 ± 0.89
	*r* ^2^	0.988

**Table 6:** Comparison of the adsorption capacity of organobentonite with various adsorbents.

Adsorbent	*Q*_max_ (mg/g)	Reference
Polyaniline-sawdust composite (PAni/SD)	2.45	[76]
Sawdust	4.71	[77]
Chitin	30.00	[78]
Organobentonite (HDTMA-Bent)	40.79	This study
Organobentonite (1CEC-NaB)	43.3	[41]
Carbon nanotubes (CNTs)	52.24	[79]
Hen feather	64.1	[80]
Nigerian soil	83.33	[81]
Cross-linked chitosan-coated bentonite (CCB)	294.1	[82]

## Data Availability

The data that support the characterization results of the adsorbent (XRD, FT-IR, and N2 adsorption/desorption) and the batch adsorption experiments appear in the document in the form of figures and tables. All the data are available.
